# 1-[2-(2,4-Dichloro­benz­yloxy)-2-(furan-2-yl)eth­yl]-1*H*-benzotriazole

**DOI:** 10.1107/S1600536811053104

**Published:** 2011-12-17

**Authors:** Özden Özel Güven, Meral Bayraktar, Simon J. Coles, Tuncer Hökelek

**Affiliations:** a Department of Chemistry, Zonguldak Karaelmas University, 67100 Zonguldak, Turkey; b Department of Chemistry, Southampton University, SO17 1BJ Southampton, England; c Department of Physics, Hacettepe University, 06800 Beytepe, Ankara, Turkey

## Abstract

In the title compound, C_19_H_15_Cl_2_N_3_O_2_, the benzotriazole ring system is approximately planar [maximum deviation = 0.018 (2) Å] and its mean plane is oriented at dihedral angles of 30.70 (5) and 87.38 (4)°, respectively, to the furan and benzene rings while the dihedral angle between furan and benzene rings is 74.46 (6)°. In the crystal, weak C—H⋯N hydrogen bonds link the mol­ecules into chains along the *b* axis. π–π stacking inter­actions between the parallel dichloro­benzene rings of adjacent mol­ecules [centroid–centroid distance = 3.6847 (9) Å] and weak C—H⋯π inter­actions are also observed.

## Related literature

For general background to the biological activity of benzotriazole derivatives, see: Hirokawa *et al.* (1998 ▶); Yu *et al.* (2003 ▶); Kopanska *et al.* (2004 ▶); Özel Güven *et al.* (2007*a* ▶,*b* ▶); Peeters *et al.* (1979 ▶); Freer *et al.* (1986 ▶). For related structures, see: Özel Güven *et al.* (2008 ▶, 2009 ▶, 2010*a* ▶,*b* ▶, 2011 ▶. For the synthesis of 2-(1*H*-benzotriazol-1-yl)-1-(furan-2-yl)ethanol, see: Özel Güven *et al.* (2012 ▶).
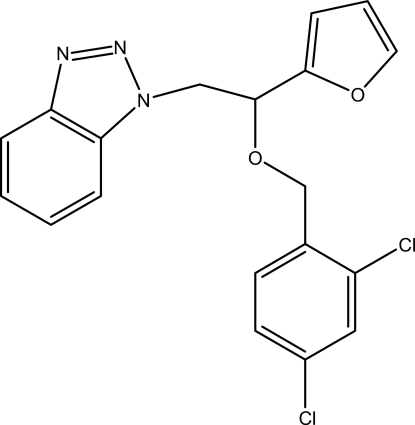


## Experimental

### 

#### Crystal data

C_19_H_15_Cl_2_N_3_O_2_
*M* *_r_* = 388.24
Monoclinic, 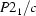

*a* = 11.5452 (2) Å
*b* = 20.0350 (5) Å
*c* = 8.3317 (2) Å
β = 105.598 (2)°
*V* = 1856.21 (7) Å^3^
*Z* = 4
Mo *K*α radiation
μ = 0.37 mm^−1^
*T* = 120 K
0.50 × 0.30 × 0.08 mm

#### Data collection

Bruker–Nonius KappaCCD diffractometer
Absorption correction: multi-scan (*SADABS*; Sheldrick, 2007 ▶) *T*_min_ = 0.837, *T*_max_ = 0.971
31461 measured reflections
4251 independent reflections
3252 reflections with *I* > 2σ(*I*)
*R*_int_ = 0.061

#### Refinement

*R*[*F* ^2^ > 2σ(*F* ^2^)] = 0.040
*wR*(*F* ^2^) = 0.103
*S* = 1.04
4251 reflections
235 parameters
H-atom parameters constrained
Δρ_max_ = 0.23 e Å^−3^
Δρ_min_ = −0.34 e Å^−3^

### 

Data collection: *COLLECT* (Nonius, 1998 ▶); cell refinement: *DENZO* (Otwinowski & Minor, 1997 ▶) and *COLLECT*; data reduction: *DENZO* and *COLLECT*; program(s) used to solve structure: *SHELXS97* (Sheldrick, 2008 ▶); program(s) used to refine structure: *SHELXL97* (Sheldrick, 2008 ▶); molecular graphics: *ORTEP-3 for Windows* (Farrugia, 1997 ▶); software used to prepare material for publication: *WinGX* (Farrugia, 1999 ▶) and *PLATON* (Spek, 2009 ▶).

## Supplementary Material

Crystal structure: contains datablock(s) I, global. DOI: 10.1107/S1600536811053104/xu5406sup1.cif

Structure factors: contains datablock(s) I. DOI: 10.1107/S1600536811053104/xu5406Isup2.hkl

Supplementary material file. DOI: 10.1107/S1600536811053104/xu5406Isup3.cml

Additional supplementary materials:  crystallographic information; 3D view; checkCIF report

## Figures and Tables

**Table 1:** Hydrogen-bond geometry (Å, °) *Cg* is the centroid of the C14–C19 ring.

*D*—H⋯*A*	*D*—H	H⋯*A*	*D*⋯*A*	*D*—H⋯*A*
C4—H4⋯N3^i^	0.93	2.59	3.452 (2)	155
C8—H8⋯*Cg*^ii^	0.93	2.92	3.782 (2)	155

Symmetry codes: (i) 
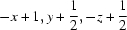
; (ii) 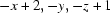.

**Fig. 1.**
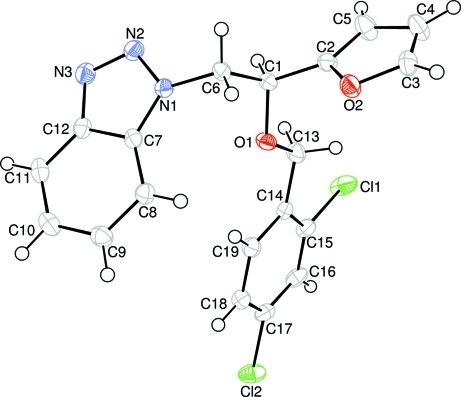
The molecular structure of the title compound with the atom-numbering scheme. Displacement ellipsoids are drawn at the 50% probability level.

**Fig. 2.**
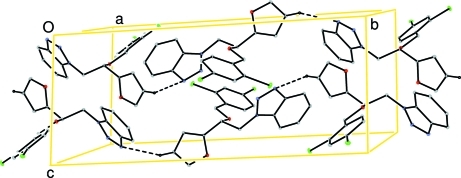
A partial packing diagram. Hydrogen bonds are shown as dashed lines. Hydrogen atoms not involved in hydrogen bonding have been omitted for clarity.

## supplementary crystallographic information

### Comment

In recent years, there has been increasing interest in syntheses of heterocyclic compounds that have biological and commercial importance. Miconazole and econazole have been developed for clinical uses as antifungal agents. Similar structures containing benzimidazole ring in place of imidazole ring of miconazole and econazole have been reported showing more antibacterial activity than antifungal activity (Özel Güven *et al.*, 2007*a*,*b*). Benzotriazole derivatives also exhibit a good degree of analgesic, anti-inflammatory, diuretic, antiviral and antihypertensive activities (Hirokawa *et al.*, 1998; Yu *et al.*, 2003; Kopanska *et al.*, 2004). The crystal structures of miconazole (Peeters *et al.*, 1979), econazole (Freer *et al.*, 1986) and similar ether compounds (Özel Güven *et al.*, 2008;  Özel Güven *et al.*, 2009; Özel Güven *et al.*, 2010*a*,*b*; Özel Güven *et al.*, 2011) have been reported, previously. Now, we report herein the crystal structure of a new benzotriazole derivative, (I).

In the molecule of the title compound (Fig. 1), the bond lengths and angles are generally within normal ranges. The benzotriazole [B (N1-N3/C7-C12)] ring system is approximately planar with a maximum deviation of 0.018 (2) Å for atom C9 and its mean plane is oriented with respect to the furan [A (O2/C2-C5)] and benzene [C (C14-C19)] rings at dihedral angles of A/B = 30.70 (5) and B/C = 87.38 (4) °. The dihedral angle between furan and benzene rings is A/C = 74.46 (6)°. Atom C6 is -0.033 (2) Å away from the plane of the benzotriazole ring and atom C1 is 0.050 (2) Å away from the plane of the furan ring, while atoms Cl1, Cl2, O1 and C13 are 0.0309 (5), 0.0223 (5), 0.0817 (11) and 0.0195 (18) Å away from the plane of the benzene ring, respectively.

In the crystal, weak C—H···N hydrogen bonds (Table 1) link the molecules into chains along the b-axis (Fig. 2). There also exists a π–π contact between the benzene rings, Cg4—Cg4^i^, may further stabilize the structure [centroid-centroid distance = 3.685 (1) Å; symmetry code: (i) 2 - x, -y, -z; Cg4 is the centroid of the ring C (C14-C19)]. A weak C—H···π interaction (Table 1) may stabilize the structure.

### Experimental

The title compound, (I), was synthesized by the reaction of 2-(1*H*-benzotriazol-1-yl)-1-(furan-2-yl)ethanol (Özel Güven *et al.*, 2012) with aryl halide using NaH. 2-(1*H*-Benzotriazol-1-yl)-1-(furan-2-yl)ethanol (219 mg, 0.95 mmol) was dissolved in DMF (4 ml). NaH (38 mg, 0.96 mmol) was added to the solution portionwise. After stirring the mixture a few minutes, 2,4-dichlorobenzyl bromide (229 mg, 0.95 mmol) was added dropwise. Then, the reaction mixture was stirred additional 3 h at room temperature. Adding methanol (5 ml), the reaction was stopped. After evaporation of the solvent, dichloromethane was added to the reaction mixture and extracted with water. Then, the organic phase was separated, dried, filtered and evaporated. The precipitate formed was purified by column chromatography using chloroform and crystallized from 2-propanol to obtain colorless crystals suitable for X-ray analysis (yield; 295 mg, 80%).

### Refinement

H atoms were positioned geometrically with C—H = 0.98, 0.93 and 0.97 Å for methine, aromatic and methylene H, respectively, and constrained to ride on their parent atoms, with *U*_iso_(H) = 1.2 *U*_eq_(C).

### Crystal data

**Table:**

C_19_H_15_Cl_2_N_3_O_2_	*F*(000) = 800
*M**_r_* = 388.24	*D*_x_ = 1.389 Mg m^−^^3^
Monoclinic, *P*2_1_/*c*	Mo *K*α radiation, λ = 0.71073 Å
Hall symbol: -P 2ybc	Cell parameters from 11553 reflections
*a* = 11.5452 (2) Å	θ = 2.9–27.5°
*b* = 20.0350 (5) Å	µ = 0.37 mm^−^^1^
*c* = 8.3317 (2) Å	*T* = 120 K
β = 105.598 (2)°	Slab, colorless
*V* = 1856.21 (7) Å^3^	0.50 × 0.30 × 0.08 mm
*Z* = 4	

### Data collection

**Table:**

Bruker–Nonius KappaCCD diffractometer	4251 independent reflections
Radiation source: fine-focus sealed tube	3252 reflections with *I* > 2σ(*I*)
graphite	*R*_int_ = 0.061
φ and ω scans	θ_max_ = 27.6°, θ_min_ = 3.3°
Absorption correction: multi-scan (*SADABS*; Sheldrick, 2007)	*h* = −14→14
*T*_min_ = 0.837, *T*_max_ = 0.971	*k* = −26→26
31461 measured reflections	*l* = −10→10

### Refinement

**Table:**

Refinement on *F*^2^	Primary atom site location: structure-invariant direct methods
Least-squares matrix: full	Secondary atom site location: difference Fourier map
*R*[*F*^2^ > 2σ(*F*^2^)] = 0.040	Hydrogen site location: inferred from neighbouring sites
*wR*(*F*^2^) = 0.103	H-atom parameters constrained
*S* = 1.04	*w* = 1/[σ^2^(*F*_o_^2^) + (0.0497*P*)^2^ + 0.507*P*] where *P* = (*F*_o_^2^ + 2*F*_c_^2^)/3
4251 reflections	(Δ/σ)_max_ < 0.001
235 parameters	Δρ_max_ = 0.23 e Å^−^^3^
0 restraints	Δρ_min_ = −0.34 e Å^−^^3^

### Special details

**Table:**

Geometry. All esds (except the esd in the dihedral angle between two l.s. planes) are estimated using the full covariance matrix. The cell esds are taken into account individually in the estimation of esds in distances, angles and torsion angles; correlations between esds in cell parameters are only used when they are defined by crystal symmetry. An approximate (isotropic) treatment of cell esds is used for estimating esds involving l.s. planes.
Refinement. Refinement of F^2^ against ALL reflections. The weighted R-factor wR and goodness of fit S are based on F^2^, conventional R-factors R are based on F, with F set to zero for negative F^2^. The threshold expression of F^2^ > 2sigma(F^2^) is used only for calculating R-factors(gt) etc. and is not relevant to the choice of reflections for refinement. R-factors based on F^2^ are statistically about twice as large as those based on F, and R- factors based on ALL data will be even larger.

### Fractional atomic coordinates and isotropic or equivalent isotropic displacement parameters (Å^2^)

**Table:**

	*x*	*y*	*z*	*U*_iso_*/*U*_eq_	
Cl1	0.89226 (4)	0.18886 (2)	0.01379 (7)	0.04089 (16)	
Cl2	1.28141 (4)	0.02933 (3)	0.12097 (6)	0.03513 (14)	
O1	0.75307 (10)	0.03838 (5)	0.30260 (14)	0.0220 (3)	
O2	0.66612 (11)	0.09918 (6)	0.57356 (14)	0.0268 (3)	
N1	0.60882 (12)	−0.07713 (7)	0.26337 (16)	0.0203 (3)	
N2	0.51947 (12)	−0.09229 (7)	0.12492 (17)	0.0243 (3)	
N3	0.55075 (13)	−0.14513 (7)	0.05429 (18)	0.0256 (3)	
C1	0.62928 (14)	0.04521 (8)	0.3022 (2)	0.0207 (3)	
H1	0.5806	0.0518	0.1873	0.025*	
C2	0.60997 (14)	0.10283 (8)	0.4057 (2)	0.0210 (3)	
C3	0.63944 (16)	0.15769 (9)	0.6416 (2)	0.0291 (4)	
H3	0.6660	0.1687	0.7540	0.035*	
C4	0.57008 (18)	0.19676 (9)	0.5242 (2)	0.0335 (4)	
H4	0.5407	0.2388	0.5395	0.040*	
C5	0.55017 (17)	0.16100 (9)	0.3709 (2)	0.0320 (4)	
H5	0.5046	0.1752	0.2668	0.038*	
C6	0.59430 (14)	−0.02090 (8)	0.3666 (2)	0.0209 (3)	
H6A	0.6437	−0.0281	0.4794	0.025*	
H6B	0.5111	−0.0186	0.3701	0.025*	
C7	0.70139 (14)	−0.12118 (8)	0.28229 (19)	0.0190 (3)	
C8	0.81400 (14)	−0.12713 (8)	0.3997 (2)	0.0227 (3)	
H8	0.8386	−0.0980	0.4894	0.027*	
C9	0.88576 (16)	−0.17884 (8)	0.3736 (2)	0.0258 (4)	
H9	0.9616	−0.1843	0.4470	0.031*	
C10	0.84755 (16)	−0.22394 (8)	0.2385 (2)	0.0275 (4)	
H10	0.8986	−0.2584	0.2265	0.033*	
C11	0.73701 (16)	−0.21804 (8)	0.1249 (2)	0.0266 (4)	
H11	0.7121	−0.2478	0.0367	0.032*	
C12	0.66331 (15)	−0.16504 (8)	0.1477 (2)	0.0214 (3)	
C13	0.79095 (15)	0.09078 (8)	0.2133 (2)	0.0251 (4)	
H13A	0.7933	0.1326	0.2729	0.030*	
H13B	0.7345	0.0956	0.1043	0.030*	
C14	0.91411 (14)	0.07481 (8)	0.1944 (2)	0.0207 (3)	
C15	0.96919 (15)	0.11711 (8)	0.1043 (2)	0.0250 (4)	
C16	1.08124 (15)	0.10456 (9)	0.0812 (2)	0.0269 (4)	
H16	1.1158	0.1338	0.0207	0.032*	
C17	1.14060 (14)	0.04695 (9)	0.1510 (2)	0.0242 (4)	
C18	1.09010 (15)	0.00307 (8)	0.2414 (2)	0.0238 (4)	
H18	1.1309	−0.0354	0.2874	0.029*	
C19	0.97711 (15)	0.01756 (8)	0.2619 (2)	0.0228 (4)	
H19	0.9427	−0.0118	0.3223	0.027*	

### Atomic displacement parameters (Å^2^)

**Table:**

	*U*^11^	*U*^22^	*U*^33^	*U*^12^	*U*^13^	*U*^23^
Cl1	0.0253 (3)	0.0312 (3)	0.0594 (3)	−0.00467 (18)	−0.0003 (2)	0.0213 (2)
Cl2	0.0204 (2)	0.0536 (3)	0.0334 (3)	−0.00010 (18)	0.01082 (18)	0.0020 (2)
O1	0.0185 (6)	0.0211 (6)	0.0288 (6)	0.0031 (4)	0.0106 (5)	0.0030 (5)
O2	0.0287 (7)	0.0240 (6)	0.0246 (6)	0.0066 (5)	0.0017 (5)	−0.0029 (5)
N1	0.0196 (7)	0.0203 (7)	0.0211 (7)	−0.0007 (5)	0.0057 (5)	−0.0026 (5)
N2	0.0227 (7)	0.0253 (7)	0.0242 (7)	−0.0021 (6)	0.0053 (6)	−0.0028 (6)
N3	0.0268 (8)	0.0248 (7)	0.0251 (8)	−0.0028 (6)	0.0070 (6)	−0.0032 (6)
C1	0.0183 (8)	0.0209 (8)	0.0231 (8)	0.0027 (6)	0.0061 (6)	−0.0021 (7)
C2	0.0182 (8)	0.0234 (8)	0.0207 (8)	0.0019 (6)	0.0043 (6)	−0.0007 (7)
C3	0.0329 (10)	0.0261 (9)	0.0274 (9)	0.0007 (7)	0.0066 (8)	−0.0091 (7)
C4	0.0404 (11)	0.0246 (9)	0.0357 (11)	0.0114 (8)	0.0107 (9)	−0.0035 (8)
C5	0.0408 (11)	0.0299 (10)	0.0229 (9)	0.0132 (8)	0.0041 (8)	0.0007 (8)
C6	0.0209 (8)	0.0208 (8)	0.0228 (8)	0.0010 (6)	0.0092 (6)	−0.0038 (7)
C7	0.0219 (8)	0.0166 (8)	0.0205 (8)	−0.0010 (6)	0.0093 (6)	0.0019 (6)
C8	0.0240 (9)	0.0208 (8)	0.0228 (8)	−0.0010 (6)	0.0057 (7)	0.0001 (7)
C9	0.0243 (9)	0.0244 (9)	0.0290 (9)	0.0029 (7)	0.0073 (7)	0.0045 (7)
C10	0.0328 (10)	0.0222 (9)	0.0310 (10)	0.0056 (7)	0.0147 (8)	0.0033 (7)
C11	0.0363 (10)	0.0190 (8)	0.0274 (9)	−0.0010 (7)	0.0136 (8)	−0.0027 (7)
C12	0.0246 (9)	0.0196 (8)	0.0211 (8)	−0.0030 (6)	0.0079 (7)	−0.0011 (7)
C13	0.0263 (9)	0.0172 (8)	0.0336 (10)	0.0007 (6)	0.0112 (7)	0.0038 (7)
C14	0.0199 (8)	0.0186 (8)	0.0231 (8)	−0.0032 (6)	0.0047 (6)	−0.0034 (6)
C15	0.0224 (9)	0.0235 (9)	0.0260 (9)	−0.0042 (7)	0.0010 (7)	0.0037 (7)
C16	0.0229 (9)	0.0326 (9)	0.0251 (9)	−0.0103 (7)	0.0060 (7)	0.0035 (7)
C17	0.0179 (8)	0.0336 (9)	0.0211 (8)	−0.0049 (7)	0.0054 (6)	−0.0052 (7)
C18	0.0226 (8)	0.0238 (8)	0.0249 (9)	0.0004 (7)	0.0063 (7)	−0.0022 (7)
C19	0.0234 (9)	0.0203 (8)	0.0254 (9)	−0.0016 (6)	0.0077 (7)	0.0017 (7)

### Geometric parameters (Å, °)

**Table:**

Cl1—C15	1.7504 (17)	C7—C8	1.406 (2)
Cl2—C17	1.7461 (17)	C7—C12	1.399 (2)
O1—C1	1.4348 (18)	C8—C9	1.380 (2)
O1—C13	1.4220 (19)	C8—H8	0.9300
O2—C2	1.376 (2)	C9—H9	0.9300
O2—C3	1.373 (2)	C10—C9	1.418 (3)
N1—C6	1.454 (2)	C10—H10	0.9300
N1—C7	1.362 (2)	C11—C10	1.375 (2)
N2—N1	1.3591 (18)	C11—H11	0.9300
N2—N3	1.3084 (19)	C12—C11	1.405 (2)
N3—C12	1.382 (2)	C13—H13A	0.9700
C1—C2	1.493 (2)	C13—H13B	0.9700
C1—C6	1.524 (2)	C14—C13	1.506 (2)
C1—H1	0.9800	C14—C15	1.394 (2)
C2—C5	1.346 (2)	C14—C19	1.393 (2)
C3—C4	1.338 (3)	C15—C16	1.381 (2)
C3—H3	0.9300	C16—H16	0.9300
C4—H4	0.9300	C17—C16	1.388 (2)
C5—C4	1.429 (3)	C17—C18	1.384 (2)
C5—H5	0.9300	C18—H18	0.9300
C6—H6A	0.9700	C19—C18	1.391 (2)
C6—H6B	0.9700	C19—H19	0.9300
			
C13—O1—C1	112.00 (12)	C8—C9—C10	122.12 (17)
C3—O2—C2	106.11 (13)	C8—C9—H9	118.9
N2—N1—C6	119.55 (13)	C10—C9—H9	118.9
N2—N1—C7	110.26 (13)	C9—C10—H10	119.2
C7—N1—C6	130.18 (13)	C11—C10—C9	121.67 (16)
N3—N2—N1	108.92 (13)	C11—C10—H10	119.2
N2—N3—C12	108.15 (13)	C10—C11—C12	117.10 (16)
O1—C1—C2	112.06 (13)	C10—C11—H11	121.4
O1—C1—C6	105.93 (12)	C12—C11—H11	121.4
O1—C1—H1	108.9	N3—C12—C7	108.39 (14)
C2—C1—C6	111.94 (13)	N3—C12—C11	130.82 (15)
C2—C1—H1	108.9	C7—C12—C11	120.78 (15)
C6—C1—H1	108.9	O1—C13—C14	109.25 (13)
O2—C2—C1	116.31 (13)	O1—C13—H13A	109.8
C5—C2—O2	109.84 (14)	O1—C13—H13B	109.8
C5—C2—C1	133.82 (16)	C14—C13—H13A	109.8
O2—C3—H3	124.6	C14—C13—H13B	109.8
C4—C3—O2	110.77 (16)	H13A—C13—H13B	108.3
C4—C3—H3	124.6	C15—C14—C13	120.49 (15)
C3—C4—C5	106.32 (15)	C19—C14—C13	122.53 (15)
C3—C4—H4	126.8	C19—C14—C15	116.98 (15)
C5—C4—H4	126.8	C14—C15—Cl1	118.63 (13)
C2—C5—C4	106.96 (16)	C16—C15—Cl1	118.37 (13)
C2—C5—H5	126.5	C16—C15—C14	122.99 (16)
C4—C5—H5	126.5	C15—C16—C17	117.93 (15)
N1—C6—C1	112.43 (13)	C15—C16—H16	121.0
N1—C6—H6A	109.1	C17—C16—H16	121.0
N1—C6—H6B	109.1	C16—C17—Cl2	118.82 (13)
C1—C6—H6A	109.1	C18—C17—Cl2	119.61 (14)
C1—C6—H6B	109.1	C18—C17—C16	121.57 (16)
H6A—C6—H6B	107.9	C17—C18—C19	118.72 (16)
N1—C7—C12	104.27 (14)	C17—C18—H18	120.6
N1—C7—C8	133.26 (15)	C19—C18—H18	120.6
C12—C7—C8	122.47 (15)	C14—C19—H19	119.1
C7—C8—H8	122.1	C18—C19—C14	121.81 (16)
C9—C8—C7	115.84 (15)	C18—C19—H19	119.1
C9—C8—H8	122.1		
			
C13—O1—C1—C2	−69.97 (17)	N1—C7—C12—N3	0.40 (17)
C13—O1—C1—C6	167.70 (13)	N1—C7—C8—C9	−178.39 (17)
C1—O1—C13—C14	−170.80 (13)	C12—C7—C8—C9	0.5 (2)
C3—O2—C2—C1	177.97 (14)	N1—C7—C12—C11	179.67 (15)
C3—O2—C2—C5	−0.38 (19)	C8—C7—C12—N3	−178.76 (14)
C2—O2—C3—C4	0.0 (2)	C8—C7—C12—C11	0.5 (2)
N2—N1—C6—C1	−84.26 (17)	C7—C8—C9—C10	−1.1 (2)
C7—N1—C6—C1	96.81 (19)	C11—C10—C9—C8	0.7 (3)
N2—N1—C7—C8	178.45 (16)	C12—C11—C10—C9	0.3 (2)
N2—N1—C7—C12	−0.58 (17)	N3—C12—C11—C10	178.19 (16)
C6—N1—C7—C8	−2.5 (3)	C7—C12—C11—C10	−0.9 (2)
C6—N1—C7—C12	178.42 (15)	C15—C14—C13—O1	177.03 (15)
N3—N2—N1—C6	−178.57 (13)	C19—C14—C13—O1	−1.8 (2)
N3—N2—N1—C7	0.56 (17)	C13—C14—C15—Cl1	−0.1 (2)
N1—N2—N3—C12	−0.28 (17)	C13—C14—C15—C16	−179.17 (16)
N2—N3—C12—C7	−0.08 (18)	C19—C14—C15—Cl1	178.80 (13)
N2—N3—C12—C11	−179.25 (17)	C19—C14—C15—C16	−0.3 (3)
O1—C1—C2—O2	−63.48 (18)	C13—C14—C19—C18	179.09 (15)
O1—C1—C2—C5	114.4 (2)	C15—C14—C19—C18	0.2 (2)
C6—C1—C2—O2	55.36 (18)	Cl1—C15—C16—C17	−178.90 (13)
C6—C1—C2—C5	−126.8 (2)	C14—C15—C16—C17	0.2 (3)
O1—C1—C6—N1	−59.57 (16)	Cl2—C17—C16—C15	179.06 (13)
C2—C1—C6—N1	178.02 (13)	C18—C17—C16—C15	0.0 (3)
O2—C2—C5—C4	0.6 (2)	Cl2—C17—C18—C19	−179.11 (12)
C1—C2—C5—C4	−177.35 (18)	C16—C17—C18—C19	0.0 (3)
O2—C3—C4—C5	0.4 (2)	C14—C19—C18—C17	−0.1 (3)
C2—C5—C4—C3	−0.6 (2)		

### Hydrogen-bond geometry (Å, °)

**Table:**

Cg is the centroid of the C14–C19 ring.

**Table:**

*D*—H···*A*	*D*—H	H···*A*	*D*···*A*	*D*—H···*A*
C4—H4···N3^i^	0.93	2.59	3.452 (2)	155
C8—H8···Cg^ii^	0.93	2.92	3.782 (2)	155

Symmetry codes: (i) −*x*+1, *y*+1/2, −*z*+1/2; (ii) −*x*+2, −*y*, −*z*+1.
